# A deep learning method for lincRNA detection using auto-encoder algorithm

**DOI:** 10.1186/s12859-017-1922-3

**Published:** 2017-12-06

**Authors:** Ning Yu, Zeng Yu, Yi Pan

**Affiliations:** 1 Department of Computing Sciences, The College at Brockport, State University of New York, 350 New Campus Drive, Brockport, 14420 NY USA; 2 School of Information Science and Technology, Southwest Jiaotong University, Chengdu, 610031 Sihuan China; 3 Department of Computer Science, Georgia State University, 25 Park Place, Atlanta, 30303 GA USA

**Keywords:** Deep learning, Long intergenic non-coding RNA (lincRNA), Auto-encoder, Transcription sites, RNA-seq, Knowledge-based discovery

## Abstract

**Background:**

RNA sequencing technique (RNA-seq) enables scientists to develop novel data-driven methods for discovering more unidentified lincRNAs. Meantime, knowledge-based technologies are experiencing a potential revolution ignited by the new deep learning methods. By scanning the newly found data set from RNA-seq, scientists have found that: (1) the expression of lincRNAs appears to be regulated, that is, the relevance exists along the DNA sequences; (2) lincRNAs contain some conversed patterns/motifs tethered together by non-conserved regions. The two evidences give the reasoning for adopting knowledge-based deep learning methods in lincRNA detection. Similar to coding region transcription, non-coding regions are split at transcriptional sites. However, regulatory RNAs rather than message RNAs are generated. That is, the transcribed RNAs participate the biological process as regulatory units instead of generating proteins. Identifying these transcriptional regions from non-coding regions is the first step towards lincRNA recognition.

**Results:**

The auto-encoder method achieves 100% and 92.4% prediction accuracy on transcription sites over the putative data sets. The experimental results also show the excellent performance of predictive deep neural network on the lincRNA data sets compared with support vector machine and traditional neural network. In addition, it is validated through the newly discovered lincRNA data set and one unreported transcription site is found by feeding the whole annotated sequences through the deep learning machine, which indicates that deep learning method has the extensive ability for lincRNA prediction.

**Conclusions:**

The transcriptional sequences of lincRNAs are collected from the annotated human DNA genome data. Subsequently, a two-layer deep neural network is developed for the lincRNA detection, which adopts the auto-encoder algorithm and utilizes different encoding schemes to obtain the best performance over intergenic DNA sequence data. Driven by those newly annotated lincRNA data, deep learning methods based on auto-encoder algorithm can exert their capability in knowledge learning in order to capture the useful features and the information correlation along DNA genome sequences for lincRNA detection. As our knowledge, this is the first application to adopt the deep learning techniques for identifying lincRNA transcription sequences.

## Background

LincRNA refers to long intergenic non-coding RNA with the length greater than 200 nucleotides that are transcribed from non-coding DNA sequences between protein-coding regions. These intergenic regions were referred as junk DNA, however, now it is discovered that intergenic regions can be transcribed and provide functional non-coding RNA genes within intergenic regions [1]. Various classes of transposable elements are embeded in lincRNAs and lincRNAs are viewed as a tool box of elements with some regulatory functions in transcription and translation. For example some lincRNAs attach to messenger RNA to block protein production [2] and families of transposable elements-derived lincRNAs have been implicated in the regulation of pluripotency [3]. In addition, lincRNA is highly tissue-specific, indicating that it might be closely related to epigenetic regulation. Thus, identifying these lincRNAs is the critical step towards understanding complicated regulatory mechanisms.

Non-coding RNA regions are four times longer than coding RNA sequences. However, currently only 21 thousand lincRNAs (about 2M bytes) are computationally discovered [4]. This is also one of the most important findings in lincRNA identification. The latest work are mostly based on RNA-seq data and heavily rely on the RNA-seq assembly technology [4, 5]. As the long intergenic non-coding RNAs are differentially expressed in different tissues and multiple conditions, the RNA-seq data sets allow to detect both rare and tissue-specific transcription events that would be undetectable in other limited studies, such as tiling array studies [6]. Thus, it establishes a philosophy that RNA-seq data can be used for lincRNA detection as the large volume of sequencing data are comprehensive and detailed. A general procedure of the state-of-the-art method to identify lincRNA is composed of the following main steps [5]: (1) Acquiring RNA-seq data set, (2) *De novo* RNA-seq assembly, (3) filtering and expression analysis.

Acquiring RNA-seq data sets is to collect the RNA sequencing data of different tissues under multiple conditions. Single RNA-seq data set cannot be used for the evidence of lincRNA detection. For example, in [4], more than one hundred previously published RNA-seq data sets covering more than twenty human tissues under multiple conditions and consisting of about four billion uniquely mapped reads. Subsequently, *De novo* RNA-seq transcriptome assembly [7] is used as the key technology to discover novel lincRNAs in a currently adopted model, which creates a transcriptome without the use of a reference genome. On the contrary, although the reference-based assembly method is a robust way of identifying transcript sequences using genome alignment, it is not able to account for incidents of structural alterations of mRNA transcripts, such as rare splicing sites and alternative splicing [8]. Instead, spliced variants are not actual proteins and they do not align continuously along the genome. An assembled transcript can be represented as introns and exons that are characterized as one of the features of an lincRNA. Thus, finding the alternative splicing transcripts from RNA-seq is regarded as one of the most important factors to the detection of novel lincRNAs. From the assembled transcripts, all known genes, pseudogenes, short ncRNAs, novel protein coding transcripts, novel UTRs, and non-lincRNA non-coding RNAs must be filtered to identify actual lincRNAs. Only intergenic non-coding transcripts with at least 200 nucleotides in length and expressed at least at one copy per cell are kept as ultimately annotated lincRNAs. A set of filters can be designed to achieve this goal.

The aforementioned techniques ensure the quality of annotated lincRNA data and provide the probability to develop a knowledge-based discovery method, although currently knowledge-based discovery methods for identifying the lincRNA remain on the preliminary stage. Driven by the newly found data set, scientists have found some hints that can corroborate their previous speculations: (1) the expression of lincRNAs appears to be regulated, that is, the relevance exists along the DNA sequences; (2) lincRNAs contain some conversed patterns/motifs tethered together by non-conserved regions [9]. The two evidences give the reasoning for developing knowledge-based deep learning methods in lincRNA detection.

The latest findings show that the expression of lincRNAs appears to be specifically regulated, although a widely accepted concept is that the degree to which intergenic transcription is functional remains uncertain and controversial [9]. According to the reasoning that negative transcripts (non-lincRNA) should lack coherent epigenetic patterns, the evaluation of lincRNAs depends on whether lincRNAs contains epigenetic markers. The catalog of lincRNAs shows some patterns of epigenetic modification similar to protein coding genes [10, 11]. For example, activating histone markers including H3K4me3 and H3K36me3 are both significantly contained within highly expressed lincRNAs; similarly, the repressive mark H3K27me3 is significantly enriched within lowly expressed lincRNAs.

The recent studies further reveal that the majority of the lincRNAs identified display a level of conservation consistent with known functional lincRNAs. This studies was performed through a 50 *nt* window to scan the sequences for the evaluation of conserved patterns [4]. Consistent with prior studies, lincRNAs display detectable but modest conservation [12]. Thus, by taking advantage of these patterns and conservations along DNA sequence, the knowledge-based discovery systems such as deep learning can discover more unidentified lincRNAs as long as the sufficient knowledge can be acquired. Fortunately, those newly found lincRNA data are able to provide such opportunities.

The preliminary concepts of deep learning including deep neural network were proposed in mid-2000s although the ideas of deep neural network had been discussed for long time since 90s [13–15]. After that, deep learning techniques have been applied to life sciences and shown tremendous promise [16–19]. Thus, deep-learning based technologies are regarded as potential tools for computational discovery of lincRNA. Deep neural network uses complicated algorithms, such as convolution, auto-encoder and Boltzmann machine etc., to constrain the error between layers and eliminate the back-propagation problem. Relying on a multiple-layer perceptron architecture, the estimation of input data through the hidden layer can be calculated by iterative encoding-decoding processing so that the minimum difference can be achieved between the input data and the estimation.

Deep learning related methods are barely seen in the methodology of lincRNA annotation. Based on those annotated data, deep learning based methods can exert their capability in knowledge learning in order to improve the aforementioned method and discover novel lincRNAs in DNA genomes.

In this project, three goals are set. The first one is developing a deep learning method for lincRNA transcription splicing sites. Second, validating the annotated lincRNAs transcription sites and testing the performance of deep learning method by comparing with conventional methods such as support vector machine (SVM) and traditional neural network based method. Third, computationally discovering other unidentified splicing sites. For the first goal, auto-encoder method achieves 100% prediction accuracy illustrated in next section. For the second and third goal, one unreported splicing site is found during re-scanning the whole annotated human lincRNA data sets through the deep learning method.

## Methods

### Auto-encoder

**Fig. 1:**
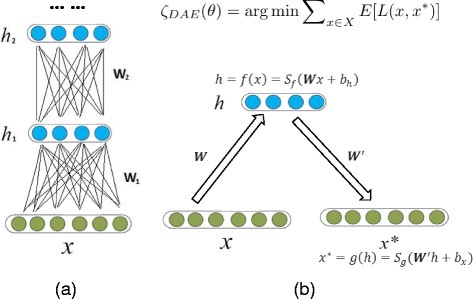
Architecture of Deep Neural Network. **a** An Illustration of Deep Neural Network Architecture. **b** An Illustration of Auto-encoder

Auto-encoder (AE) is a layer-wise training algorithm we adopt on an artificial neural network that can be used to constitute a multiple-layer percetron architectures for deep learning machine shown in Fig. 1 a. The hidden layer *h* and the iterative estimation of *x* ^∗^ can be expressed as Eq. 1 by calculating the weights as illustrated in Fig. 1 b. The iteration becomes stable when it has the minimum distance between *x* and *x* ^∗^, as shown in Eq. 2. The preliminary ideas of shallow/deep neural network had been discussed for long time since 90s, however, mature concepts of deep learning including deep neural network were proposed in mid-2000s [13–15]. Since then, it has been applied to life sciences and shown tremendous promise [16–19].

The simplest auto-encoder is based on a feedforward, non-recurrent neural network similar to the multiple-layer perceptron (MLP). The difference is that the output layer of auto-encoder has the same number of nodes as the input layer and an auto-encoder is trained to reconstruct their own inputs instead of being trained to predict the output value. Thus, training the neighboring set of two layers minimizes the errors between layers and eliminates the problem of error propagation that occurs in conventional neural network.

**Fig. 2:**
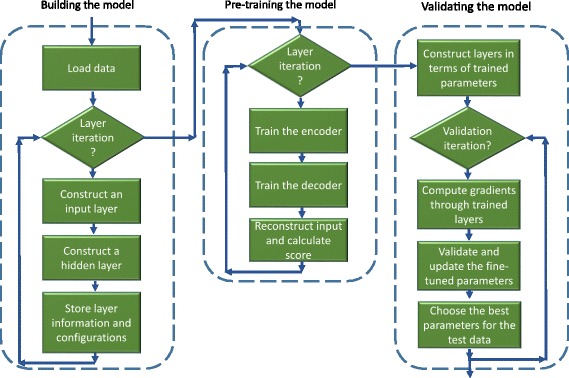
Flow Chart for Auto-encoder Method

Our auto-encoder method is composed of three main steps as shown in Fig. 2: building, pre-training and validating. In the first step, the basic architecture including input layer, hidden layer and activation functions is built; secondly, the encoder and the decoder are trained layer by layer following the pre-configured iterations; thirdly, fine-grained training/validation is performed through the entire model. In other words, the first step constructs the basic framework of the deep neural network, the second one trains the layer-wise nodes and the last one flows through all layers for validation.

As the core of auto-encoder, the pseudo-code of cost update algorithm is shown in Algorithm 1 following the Eqs. 1 and 2.

1 $$  \left\{ \begin{array}{l} h = f(x) = S_{f} (Wx + b_{h}) \\ x^{*} = g(h) = S_{g} (W'h + b_{x}) \\ \end{array} \right.  $$

2 $$  \zeta_{DAE} (\theta) = \arg \min {\sum\nolimits}_{x \in X} {E\left[L\left(x,x^{*} \right)\right]}  $$

### Transcription Sites

Similar to coding region transcription, non-coding regions are split at transcription sites. However, regulatory RNAs rather than message RNAs are generated. That is, the transcribed RNAs participate the biological process as regulatory units instead of generating proteins. Thus, identifying these transcriptional regions is the first step towards lincRNA recognition. Similar to gene structures, lincRNAs have the complicated exon/intron structures, whereas the difference from gene structures is that many of them have two exons or three exons only.

Benefiting from the increasing annotation data in lincRNAs, lincRNA transcriptional splicing site sequences are collected from the annotated human DNA genome data. However, the annotated data sets of lincRNAs are not so many as that of mRNAs. Thus, all of annotated lincRNAs are used for training, validation and testing.

In the same vein to detection of protein-coding splicing sites, auto-encoder neural network method is used for the lincRNA application. A 2-layer auto-encoder model is used for lincRNA detection and various encoding schemes are used for evaluating the best performance. The similar knowledge-based deep learning methods in lincRNA detection is barely mentioned in literature so far. The experimental results show an excellent predictive performance of deep neural network method on lincRNA data sets.

### Encoding Schemes of DNA Sequence

**Fig. 3:**
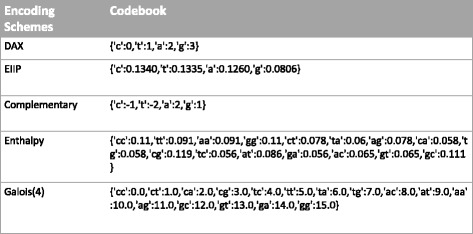
Five Encoding Schemes

Data representation, particularly the encoding scheme of DNA sequence, is one of important factors that can largely impact on the performance of knowledge-based discovery systems. Different from other data format, the DNA nucleotide sequences are recorded as human readable characters, C, T, A and G. Adopting the improper encoding schemes to feed the learning machine can lead to the failure of prediction task. The encoding schemes we test are shown as Fig. 3, including DAX [20], EIIP [21], Complementary [22], Enthalpy [23], and Galois(4) [24] schemes.

### Algorithm Implementation and Validation

The auto-encoder algorithm for lincRNA detection is implemented on open source Python libraries, Theano and Keras. The training and validation data sets including the known lincRNA data are collected from UCSC Genome Browser database. The existing methods, including NNSplice [25] and Libsvm [26], are used for validating the proposed deep learning method by the comparisons with traditional Neural Network and Support Vector Machine.

According to the latest findings [4], totally 46,983 lincRNA sequences containing 90 nucleotides and 89,287 lincRNA sequences containing 15 nucleotides are extracted and collected as transcriptional sites, Acceptors and Donors respectively, including 5,000 sequences as validation in each data set. Based on the auto-encoder algorithm, a 2-layer neural network is constructed for the experiments. Five aforementioned encoding schemes are used for comparing and acquiring the best performance.

## Results

Tables 1 and 2 respectively show the comparison results for the two data sets. It shows that 100% predictive rate of deep neural network method with complementary encoding scheme on the acceptor data, meaning that complementary scheme has the strong ability on more-feature data sets. Similar performances among all encoding schemes show the similar ability on less-feature data set.

**Table 1:** Results on lincRNA Acceptor Data

^*a*^	I	II	III	IV	V
TP	49.4	49.4	49.0	49.4	49.4
FP	0.0	0.2	0.0	1.4	50.6
FN	0.0	0.4	0.0	0.1	0.0
TN	50.5	50.4	0.6	49.2	0.0
^*b*^	I	II	III	IV	V
Sn	**100.0**	99.2	**100.0**	99.9	100.0*
Sp	99.9	99.6	**100.0**	97.2	0.0
Acc	100.0	99.4	**100.0**	98.5	49.4
Mcc	99.9	98.8	**100.0**	97.1	–
Ppv	99.9	99.6	**100.0**	97.2	49.4
Pc	99.9	98.8	**100.0**	97.1	49.4
F1	**100.0**	99.4	**100.0**	98.5	66.1

I: DAX, II: EIIP, III: Complimentary, IV: Enthalpy, V: Galois

Panel ^*a*^: the measurement of methods

TP: True positive

FP: False positive

FN: False negative

TN: True negative

Panel ^*b*^: the evaluation of methods

Sensitivity, *Sn*=*TP*/(*TP*+*FN*)

Specificity, *Sp*=*TN*/(*TN*+*FP*)

Accuracy, *Acc*=(*TP*+*TN*)/(*TP*+*FP*+*FN*+*TN*)

Matthews correlation coefficient, $Mcc=TP\times TN - FN\times FP \over {\sqrt {(TP + FN) \times (TN + FP) \times (TP + FP) \times (TN + FN)} }$

Positive predictive value, *Ppv*=*TP*/(*TP*+*FP*)

Performance coefficient, *Pc*=*TP*/(*TP*+*FN*+*FP*)

F1 score, the harmonic mean of precision and sensitivity, *F*1=2×*TP*/(2×*TP*+*FP*+*FN*)

*: Not eligible for comparison due to training failure

–: Invalid value

**Table 2:** Results on lincRNA Donor Data

^*a*^	I	II	III	IV	V
TP	7.7	9.0	8.5	11.2	0.0
FP	2.1	2.7	2.8	4.5	0.0
FN	6.7	5.4	5.9	3.2	14.4
TN	83.5	82.9	82.8	81.1	85.6
^*b*^	I	II	III	IV	V
Sn	53.2	62.5	58.8	**78.1**	0.0
Sp	**97.6**	96.9	96.7	94.8	100.0*
Acc	91.2	91.9	91.2	**92.4**	85.6
Mcc	60.1	64.9	61.5	**70.2**	–
Ppv	**78.6**	77.1	75.0	71.5	–
Pc	46.5	52.7	49.1	**59.5**	0.0
F1	63.5	69.0	65.9	**74.6**	0.0

I: DAX, II: EIIP, III: Complimentary, IV: Enthalpy, V: Galois

Panel ^*a*^: the measurement of methods

Panel ^*b*^: the evaluation of methods

*: Not eligible for comparison due to training failure

–: Invalid value

**Fig. 4:**
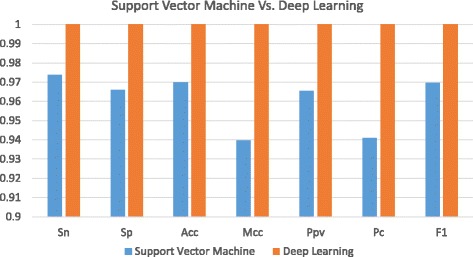
Comparison between Support Vector Machine and Deep Learning on lincRNA Acceptor Data Set

**Fig. 5:**
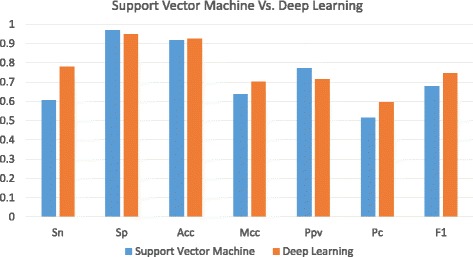
Comparison between Support Vector Machine and Deep Learning on lincRNA Donor Data Set

Moreover, we compare the deep learning method with Support Vector Machine (SVM) using the same data sets. SVM software is tested on the latest version of libsvm [26]. Figures 4 and 5 show the comparative results that auto-encoder based deep learning method has an extraordinary ability over conventional SVM method. On the data set with more features in Fig. 4, the deep learning method shows the large superiority over SVM while their performances are very close on the data set with less features in Fig. 5.

**Fig. 6:**
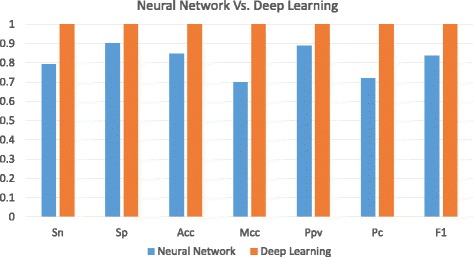
Comparison between Conventional Neural Network Method and Deep Learning Method on lincRNA Acceptor Data Set

**Fig. 7:**
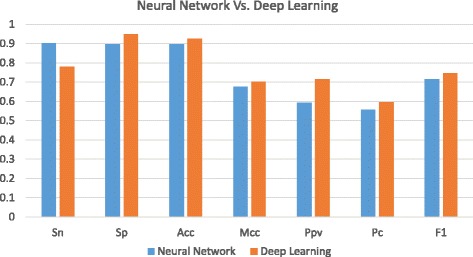
Comparison between Conventional Neural Network Method and Deep Learning Method on lincRNA Donor Data Set

In addition, a comparison between the deep learning method and the traditional neural network (NN) based method [25] is also conducted. Figures 6 and 7 show that DL outperforms the conventional NN based method for detection of transcriptional sites using lincRNA data sets. Similarly, on the data set with more features in Fig. 6, the deep learning method distinguishes itself from the NN based method while their performances are very close on the data set with less features in Fig. 7. It means that various methods have the similar performance on handling the less-feature data set while deep learning can have a large superiority over others on processing the more-feature data set. Such experimental results also manifest that deep learning based method can have better performance than other conventional methods for prediction of lincRNAs on DNA sequence data. The reason that we separate the comparison between SVM-DL group and NN-DL group is that the SVM tool we use for the experiment can accept all encoding schemes as its input while the NN-based web tool accepts only the DNA sequence as its input.

**Fig. 8:**
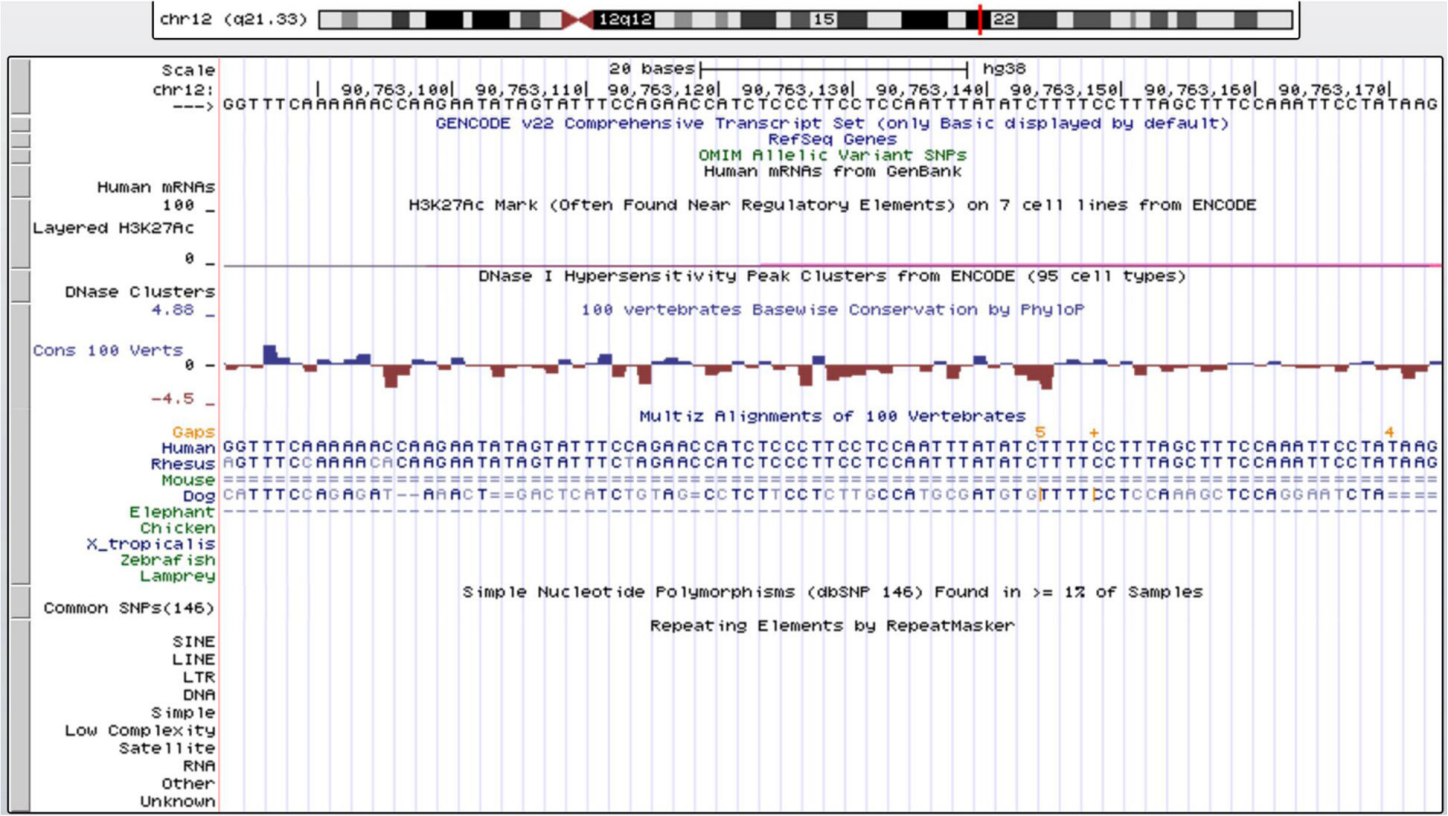
An Unidentified lincRNA Acceptor Site

Figure 8 shows an unreported splicing site is found by re-scanning the whole human genome through the deep learning method, which is located at 90,763,154 chromosome 12 (hg38) within the annotated lincRNA chr12_90761911_90806776. This result is based on the aforementioned deep learning method that was tested with 100% accuracy on acceptor data set.

## Discussion

Although a deep learning based method has been illustrated for lincRNA detection, distinguishing the coding and non-coding transcription is still an open problem because the transcribed regions have the similar structures of exon and intron in both coding and non-coding regions. Practically, it is hard to find an effective way to differentiate the two types of transcripts. Thus, the intergenic regions have to be selected and the pre-processing is necessary for detection, which is the downside of our method and partially limits the use of the proposed deep learning based method.

In addition, the development of deep learning method for lincRNA detection is still on preliminary stage and the prototype of the auto-encoder based method has more spaces to improve. For example, function modules need to be uniformed and parameters in the work flow have to be optimized.

## Conclusion

RNA-seq technologies generate a large volume of transcriptional data that scientists can utilize for lincRNA annotation. Derived from the observations from the newly found lincRNA data set, two evidences can provide the reasoning for adopting knowledge-based deep learning methods in lincRNA detection: (1) the expression of lincRNAs appears to be regulated, indicating that the relevance exists along the DNA sequences; (2) lincRNAs contain some conversed patterns/motifs tethered together by non-conserved regions [9]. In this project, a knowledge-based discovery method using the emerging deep learning technology for lincRNA detection is proposed and developed on DNA genome analysis. It takes advantage of the latest findings of lincRNA data set and aims to utilize the cutting-edge knowledge-based method, namely auto-encoder algorithm, in order to extract the features of lincRNA transcription sites in a more accurate way than conventional methods. The results show its superiority over the support vector machine and the conventional neural network based method.

In the future, developing a generic framework based on deep learning for lincRNA prediction will be focused on, which can provide an uniform platform for user interfaces. Meanwhile, the studies on lincRNA detection will be carried out on other species such as mouse and other mammals.
